# Differences in mortality in critically ill elderly patients during the second COVID-19 surge in Europe

**DOI:** 10.1186/s13054-021-03739-7

**Published:** 2021-09-23

**Authors:** Christian Jung, Jesper Fjølner, Raphael Romano Bruno, Bernhard Wernly, Antonio Artigas, Bernardo Bollen Pinto, Joerg C. Schefold, Georg Wolff, Malte Kelm, Michael Beil, Sigal Sviri, Peter Vernon van Heerden, Wojciech Szczeklik, Miroslaw Czuczwar, Michael Joannidis, Sandra Oeyen, Tilemachos Zafeiridis, Finn H. Andersen, Rui Moreno, Susannah Leaver, Ariane Boumendil, Dylan W. De Lange, Bertrand Guidet, Hans Flaatten, , COVIP Study Group, Philipp Eller, Michael Joannidis, Dieter Mesotten, Pascal Reper, Sandra Oeyen, Walter Swinnen, Helene Brix, Jens Brushoej, Maja Villefrance, Helene Korvenius Nedergaard, Anders Thais Bjerregaard, Ida Riise Balleby, Kasper Andersen, Maria Aagaard Hansen, Stine Uhrenholt, Helle Bundgaard, Jesper Fjølner, Aliae AR Hussein Mohamed, Rehab Salah, Yasmin Khairy NasrEldin Mohamed Ali, Kyrillos Wassim, Yumna A Elgazzar, Samar Tharwat, Ahmed Y Azzam, Ayman abdelmawgoad Habib, Hazem Maarouf Abosheaishaa, Mohammed A Azab, Susannah Leaver, Arnaud Galbois, Bertrand Guidet, Cyril Charron, Emmanuel Guerot, Guillaume Besch, Jean-Philippe Rigaud, Julien Maizel, Michel Djibré, Philippe Burtin, Pierre Garcon, Saad Nseir, Xavier Valette, Nica Alexandru, Nathalie Marin, Marie Vaissiere, Gaëtan Plantefeve, Thierry Vanderlinden, Igor Jurcisin, Buno Megarbane, Anais Caillard, Arnaud Valent, Marc Garnier, Sebastien Besset, Johanna Oziel, Jean-herlé Raphaelen, Stéphane Dauger, Guillaume Dumas, Bruno Goncalves, Gaël Piton, Christian Jung, Raphael Romano Bruno, Malte Kelm, Georg Wolff, Eberhard Barth, Ulrich Goebel, Eberhard Barth, Anselm Kunstein, Michael Schuster, Martin Welte, Matthias Lutz, Patrick Meybohm, Stephan Steiner, Tudor Poerner, Hendrik Haake, Stefan Schaller, Detlef Kindgen-Milles, Christian Meyer, Muhammed Kurt, Karl Friedrich Kuhn, Winfried Randerath, Jakob Wollborn, Zouhir Dindane, Hans-Joachim Kabitz, Ingo Voigt, Gonxhe Shala, Andreas Faltlhauser, Nikoletta Rovina, Zoi Aidoni, Evangelia Chrisanthopoulou, Antonios Papadogoulas, Mohan Gurjar, Ata Mahmoodpoor, Abdullah Khudhur Ahmed, Brian Marsh, Ahmed Elsaka, Sigal Sviri, Vittoria Comellini, Ahmed Rabha, Hazem Ahmed, Silvio a Namendys-Silva, Abdelilah Ghannam, Martijn Groenendijk, Marieke Zegers, Dylan de Lange, Alex Cornet, Mirjam Evers, Lenneke Haas, Tom Dormans, Willem Dieperink, Luis Romundstad, Britt Sjøbø, Finn H Andersen, Hans Frank Strietzel, Theresa Olasveengen, Michael Hahn, Miroslaw Czuczwar, Ryszard Gawda, Jakub Klimkiewicz, Maria Campos de LurdesSantos, André Gordinho, Henrique Santos, Rui Assis, Ana Isabel Pinho Oliveira, Mohamed Raafat Badawy, David Perez-Torres, Gemma Gomà, Mercedes Ibarz Villamayor, Angela Prado Mira, Patricia Jimeno Cubero, Susana Arias Rivera, Teresa Tomasa, David Iglesias, Eric Mayor Vázquez, Cesar Aldecoa, Aida Fernández Ferreira, Begoña Zalba-Etayo, Isabel Canas-Perez, Luis Tamayo-Lomas, Cristina Diaz-Rodriguez, Susana Sancho, Jesús Priego, Enas M.Y. Abualqumboz, Momin Majed Yousuf Hilles, Mahmoud Saleh, Nawfel Ben-HAmouda, Andrea Roberti, Alexander Dullenkopf, Yvan Fleury, Bernardo Bollen Pinto, Joerg C Schefold, Mohammed Al-Sadawi, Nicolas Serck, Elisabeth Dewaele, Pritpal Kumar, Camilla Bundesen, Richard Innes, James Gooch, Lenka Cagova, Elizabeth Potter, Michael Reay, Miriam Davey, Sally Humphreys, Caroline Hauw Berlemont, Benjamin Glenn Chousterman, François Dépret, Alexis Ferre, Lucie Vettoretti, Didier Thevenin, Andreas Faltlhauser, Milena Milovanovic, Philipp Simon, Marco Lorenz, Sandra Emily Stoll, Simon Dubler, Kristina Fuest, Francesk Mulita, Eumorifa Kondili, Ioannis Andrianopoulos, Iwan Meynaar, Alexander Daniel Cornet, Britt Sjøbøe, Anna Kluzik, Paweł Zatorski, Tomasz Drygalski, Wojciech Szczeklik, Joanna Solek-pastuszka, Dariusz Onichimowski, Jan Stefaniak, Karina Stefanska-Wronka, Ewa Zabul, Filipe Sousa Cardoso, Maria José Arche Banzo, Teresa Maria Tomasa-Irriguible, Ángela Prado Mira, Susana Arias-Rivera, Fernando Frutos-Vivar, Sonia Lopez-Cuenca, Pablo Ruiz de Gopegui, Nour Abidi, Ivan Chau, Richard Pugh, Sara Smuts

**Affiliations:** 1 Medical Faculty, Department of Cardiology, Pulmonology and Vascular Medicine, Heinrich-Heine-University Duesseldorf, Moorenstraße 5, 40225 Duesseldorf, Germany; 2 Department of Intensive Care, Aarhus University Hospital, Aarhus, Denmark; 3 Department of Cardiology, Paracelsus Medical University, Salzburg, Austria; 4 Department of Intensive Care Medicine, CIBER Enfermedades Respiratorias, Corporacion Sanitaria Universitaria Parc Tauli, Autonomous University of Barcelona, Sabadell, Spain; 5 Department of Acute Medicine, Geneva University Hospitals, Geneva, Switzerland; 6 Department of Intensive Care Medicine, Inselspital, Universitätsspital, University of Bern, Bern, Switzerland; 7 Department of Medical Intensive Care, Hadassah Medical Center and Faculty of Medicine, Hebrew University of Jerusalem, Jerusalem, Israel; 8 General Intensive Care Unit, Hadassah University Medical Center, Jerusalem, Israel; 9 Center for Intensive Care and Perioperative Medicine, Jagiellonian University Medical College, Krakow, Poland; 10 2nd Department of Anesthesiology and Intensive Care, Medical University of Lublin, Staszica 16, 20-081 Lublin, Poland; 11 Division of Intensive Care and Emergency Medicine, Department of Internal Medicine, Medical University Innsbruck, Innsbruck, Austria; 12 Department of Intensive Care 1K12IC, Ghent University Hospital, Ghent, Belgium; 13 Intensive Care Unit General Hospital of Larissa, Larissa, Greece; 14 Department of Anaesthesia and Intensive Care, Ålesund Hospital, Ålesund, Norway; 15 Department of Circulation and Medical Imaging, Norwegian University of Science and Technology, Trondheim, Norway; 16 Unidade de Cuidados Intensivos Neurocríticos E Trauma. Hospital de São José, Centro Hospitalar Universitário de Lisboa Central, Faculdade de Ciências Médicas de Lisboa, Nova Médical School, Lisbon, Portugal; 17 General Intensive Care, St George’s University Hospital NHS Foundation Trust, London, UK; 18 Sorbonne Universités, UPMC Univ Paris 06, INSERM, UMR_S 1136, Institut Pierre Louis d’Epidémiologie et de Santé Publique, Equipe: épidémiologie hospitalière qualité et organisation des soins, 75012 Paris, France; 19 Assistance Publique - Hôpitaux de Paris, Hôpital Saint-Antoine, service de réanimation médicale, 75012 Paris, France; 20 Department of Intensive Care Medicine, University Medical Center, University Utrecht, Utrecht, The Netherlands; 21 Department of Clinical Medicine, University of Bergen, Department of Anaestesia and Intensive Care, Haukeland University Hospital, Bergen, Norway

**Keywords:** Covid-19, Frailty, Outcome, Elderly, Pandemia, Surges

## Abstract

**Background:**

The primary aim of this study was to assess the outcome of elderly intensive care unit (ICU) patients treated during the spring and autumn COVID-19 surges in Europe.

**Methods:**

This was a prospective European observational study (the COVIP study) in ICU patients aged 70 years and older admitted with COVID-19 disease from March to December 2020 to 159 ICUs in 14 European countries. An electronic database was used to register a number of parameters including: SOFA score, Clinical Frailty Scale, co-morbidities, usual ICU procedures and survival at 90 days. The study was registered at ClinicalTrials.gov (NCT04321265).

**Results:**

In total, 2625 patients were included, 1327 from the first and 1298 from the second surge. Median age was 74 and 75 years in surge 1 and 2, respectively. SOFA score was higher in the first surge (median 6 versus 5, *p* < 0.0001). The PaO_2_/FiO_2_ ratio at admission was higher during surge 1, and more patients received invasive mechanical ventilation (78% versus 68%, *p* < 0.0001). During the first 15 days of treatment, survival was similar during the first and the second surge. Survival was lower in the second surge after day 15 and differed after 30 days (57% vs 50%) as well as after 90 days (51% vs 40%).

**Conclusion:**

An unexpected, but significant, decrease in 30-day and 90-day survival was observed during the second surge in our cohort of elderly ICU patients. The reason for this is unclear. Our main concern is whether the widespread changes in practice and treatment of COVID-19 between the two surges have contributed to this increased mortality in elderly patients. Further studies are urgently warranted to provide more evidence for current practice in elderly patients.

***Trial registration number*:**

NCT04321265, registered March 19th, 2020.

**Supplementary Information:**

The online version contains supplementary material available at 10.1186/s13054-021-03739-7.

## Introduction

The first surge of the COVID-19 pandemic between March and May 2020 affected the elderly population disproportionally. Elderly patients were over-represented both among ICU admissions and among non-survivors [1]. The hospital mortality in all ICU patients was observed to be around 40–50%, but a higher mortality was seen in the elderly and frail population [2, 3].

Thus, a robust prognostic stratification of elderly patients, many of whom had multiple co-morbidities, posed significant challenges in a disease which had not previously been encountered. In addition to established and new disease severity scores, geriatric characteristics notably frailty, co-morbidity and functional status were soon confirmed to be important prognostic factors in elderly COVID-19 patients [3–6]. Clinical studies were quickly launched focusing on potential novel treatments and the management of patients with COVID-19. These included respiratory management, such as the role of mechanical ventilation and prone positioning as well as pharmacological treatment (e.g. corticosteroids, anticoagulation and anti-inflammatory agents) [7, 8].

During the summer months of 2020, the spread of the virus declined. However, it became evident by early autumn that a second surge of the SARS-Cov-2 pandemic was imminent [9]. In contrast to the first surge, health care systems had now acquired an increased understanding of this disease. The pathophysiology of COVID-19 disease was described, and it was possible to predict the epidemiology of the disease based on modelling from the previous surge. Furthermore, at least theoretically, “test, track and isolate” was in operation and more robust measures such as “shielding” vulnerable individuals such as the elderly and frail were in place [10].

The aim of this study was to assess a possible outcome difference in the first and the second surge of the pandemic in critically ill elderly ICU patients. The hypothesis was that outcome of elderly ICU patients with COVID-19 improved during the second surge of the pandemic due to implemented changes in practice, based on experience and evidence available.

## Methods

### Design and setting

The study was a prospective observational multi-centre study of COVID-19 patients aged 70 years and older admitted to 159 ICUs in 14 European countries, called the COVIP study. Recruitment took place from 19 March to 31 December 2020. A list of collaborators is shown in Additional file 1. A map of participating ICUs is shown in Additional file 2. Recruitment in countries over the two different time periods is shown in Additional file 3. Recruitment within the individual countries in relation to the start of the study is shown in Additional file 4. The study was organised by the Very old Intensive care Patients (VIP) project [11, 12] within the European Society of Intensive Care Medicine (ESICM) which also endorsed the study (www.vipstudy.org). National coordinators were responsible for the recruitment of ICUs and for obtaining national and local ethical permission. In addition, national coordinators supervised patient recruitment. Due to variations in requirement for ethical consent, some countries could recruit patients without upfront informed consent, while others had to obtain it. The study deliberately allowed for co-enrolment of study patients to other COVID-19 studies. The study was registered on ClinicalTrials.gov (ID: NCT04321265) and adhered to the European Union General Data Privacy Regulation (GDPR) directive.

Study preparation started during the first phase of the pandemic, and recruitment commenced on 19 March 2020. Throughout the pandemic, recruitment to the COVIP study was monitored by weekly virtual steering group meetings. The first recruitment period, representing the first surge of the pandemic, was defined as from 19 March until 26 May 2020, and the second recruitment period, reflecting the second surge, from 1 September to 31 December 2020. This was also reflected by the number of ICU patients published by international registries. Each participating ICU included consecutive patients. To limit workload during the pandemic, centres were not asked to protocol a screening log. A diagnosis of COVID-19 was made based on a positive polymerase chain reaction (PCR) test.

### Study population

Eligible patients were 70 years or older with a proven diagnosis of COVID-19 and admitted to an intensive care unit (ICU). Pre-ICU triage was not part of this study. To avoid duplication caused by the transfer of a patient from one ICU to another, each patient could only be entered once into the database regardless of readmission, transfer or other reason. This resulted in a unique electronic database record for each patient. The reference date was day 1 of the first admission to an ICU. All consecutive days were numbered sequentially from the day of admission. To limit bias in the comparison of the two surges, only patients admitted to an ICU in a European country that recruited patients during both surges were included in the analysis (Additional file 5).

### Data collection

Centres collected data using the online case record form (CRF). Day one sequential organ-failure assessment (SOFA) score on admission was calculated either manually or using an online calculator in the electronic CRF as described previously [11, 12]. The PaO_2_/FiO_2_ ratio (PaO_2_/FiO_2_-index) on admission was calculated using the arterial PO_2_ [mmHg] and the F_i_O_2_ [fraction of 1] from the first arterial blood gas. ICU length of stay (LOS) was recorded in hours. As described previously, [11] the electronic CRF and database ran on a secure server set up by and stored at Aarhus University, Aarhus, Denmark.

### Frailty, comorbidities and organ support

The frailty level prior to the acute illness and hospital admission was assessed using the Clinical Frailty Scale (CFS) as published previously [12]. A detailed definition of co-morbidities and organ support can be found in Additional file 6. In addition, the place of living (habitat) was collected using the following categories: own household, household with family or caregivers, nursing home, in-patient in hospital.

### Outcome measurement

Patients were followed up until 90 days or death. The primary endpoint was survival at 30 days, the secondary endpoint survival at 90 days. Data could be retrieved either directly, from the hospital administration system or after discharge using telephone follow-up. Limitation of life-sustaining therapies such as withholding or withdrawing organ support was documented [13].

### Statistical analysis

Baseline characteristics of patients were analysed as frequencies and percentages for categorical variables and as medians and interquartile ranges (IQRs) for continuous variables. Comparisons between the two periods were evaluated using the Kruskal–Wallis test (ANOVA) for continuous variables and the χ2 or Fisher exact test for categorical variables as appropriate. Comparisons between surges were evaluated using the Wilcoxon test for continuous variables and the χ2 or Fisher exact test for categorical variables as appropriate.

Incidence of organ support and treatment limitations were estimated using cumulative incidence analysis considering ICU death and ICU discharge as competing risks. Univariate comparisons were performed using Gray’s test.

The crude overall survival up to 90 days after ICU admission was estimated by the Kaplan–Meier method and compared between groups using a log-rank test.

In order to compare survival between the two surges adjusting for patients characteristics, a Cox model was fitted including the following variables: age, sex, PaO2/FiO2 ratio, other SOFA components, frailty, BMI, habitat and comorbidities (definition in Additional file 6). Robust sandwich estimators to estimate the variance–covariance matrix of the regression coefficient estimates were used to account for clustering of patients within centres.

For continuous covariates, the martingale residuals was plotted against the covariates to assess the functional form of a covariate and eventually to detect nonlinearity.

In order to test proportional assumption, for each covariate independence between scaled Schoenfeld residuals was tested, a global test was also performed for the model as a whole [14].

As survival difference between surges was not constant over time and in order to investigate whether the survival difference was due to a change of strategies in ICU or hospitals, survival before and after day 15 (median ICU stay for patients discharged alive during the first surge) was studied separately. This was done in order to identify factors relevant for outcome earlier and later during the treatment course.

We first estimated our models on the complete data set and then used multiple imputation for participants with missing data, using predictive mean matching for continuous variables, logistic regression for binary data, and polytomous regression for (unordered) categorical data. The cumulative baseline hazard was approximated by the Nelson–Aalen estimator and included in the imputation model [15]. Hundred imputations were drawn. Cox models were estimated in each imputed datasets, and estimates were combined using Rubin's rules to give an overall estimate of parameters and corresponding variance–covariance matrix. Robust sandwich estimators to estimate the variance–covariance matrix of the regression coefficient estimates were used to account for clustering of patients within centres.

Incidence of organ support and treatment limitations were estimated using cumulative incidence analysis considering ICU death and ICU discharge as competing risks. Univariate comparisons were performed using Gray’s test.

The general statistical analysis was conceptualised during the set-up of the study at the beginning of the pandemic but has been revised during the analysis of the data obtained.

All p values were two-sided, and *p* < 0.05 was considered statistically significant. Statistical analyses were performed with R 3.2.3 software packages (R Development Core Team, Vienna, Austria).

## Results

As illustrated in Table 1, 2625 patients from European countries were included in the COVIP study during the two surges. There were nearly equal numbers of patients recruited during the first and the second surge of the pandemic. In the first surge, 4/1327 patients were lost to follow-up and in the second surge, 25/1298 patients were lost to follow-up. During these two surges, patient characteristics changed, with slightly increasing age, body mass index, and CFS. In addition, PaO_2_/FiO_2_ ratio and SOFA score were lower in patients during the second surge of the pandemic and there was an increased prevalence of diabetes mellitus and ischemic heart disease.

**Table 1:** Baseline characteristics of the study population across the two surges

	First surge (until 26 May 2020)		Second surge 1 September–31 December 2020		*p *value First surge versus second surge
Patients (*n*)	1327		1298		
*Characteristics*		Missing values (*N*)		Missing values (*N*)	
Age (years)	74 (72–78)	4	75 (72–79)	1	0.02
Sex (male sex)	74%	4	70%	1	0.04
Weight (kg)	80 (72–90)	107	80 (72–91)	45	0.30
Height (cm)	170 (165–177)	142	170 (164–177)	67	0.12
BMI (kg/m^2^)	27.5 (24.7–30.1)	154	27.6 (24.9–31.1)	77	0.03
*Clinical status*					
CFS	3 (2–4)	114	3 (2–4)	116	0.0011
SOFA	6 (3–8)	13	5 (3–7)	28	< 0.0001
PaO_2_/FiO_2_ ratio	126 (84–181)	18	104 (75–156)	63	< 0.0001
*Comorbidities*					
Diabetes mellitus (%)	31%	8	36%	10	0.0097
Ischemic heart disease (%)	20%	22	25%	17	0.0004
Chronic kidney disease (%)	15%	14	17%	14	0.22
Arterial hypertension (%)	66%	9	67%	8	0.52
Chronic pulmonary disease (%)	22%	16	23%	9	0.45
Chronic heart failure (%)	14%	22	15%	17	0.72
*Habitat categories*					
Own home	78%	0	80%	0	0.0001
Other home with family or caregivers	6%	0	6%	0	
Nursing home	4%	0	2%	0	
Hospital ward	6%	0	8%	0	
Other/Unknown	6%	0	4%	0	

**Fig. 1:**
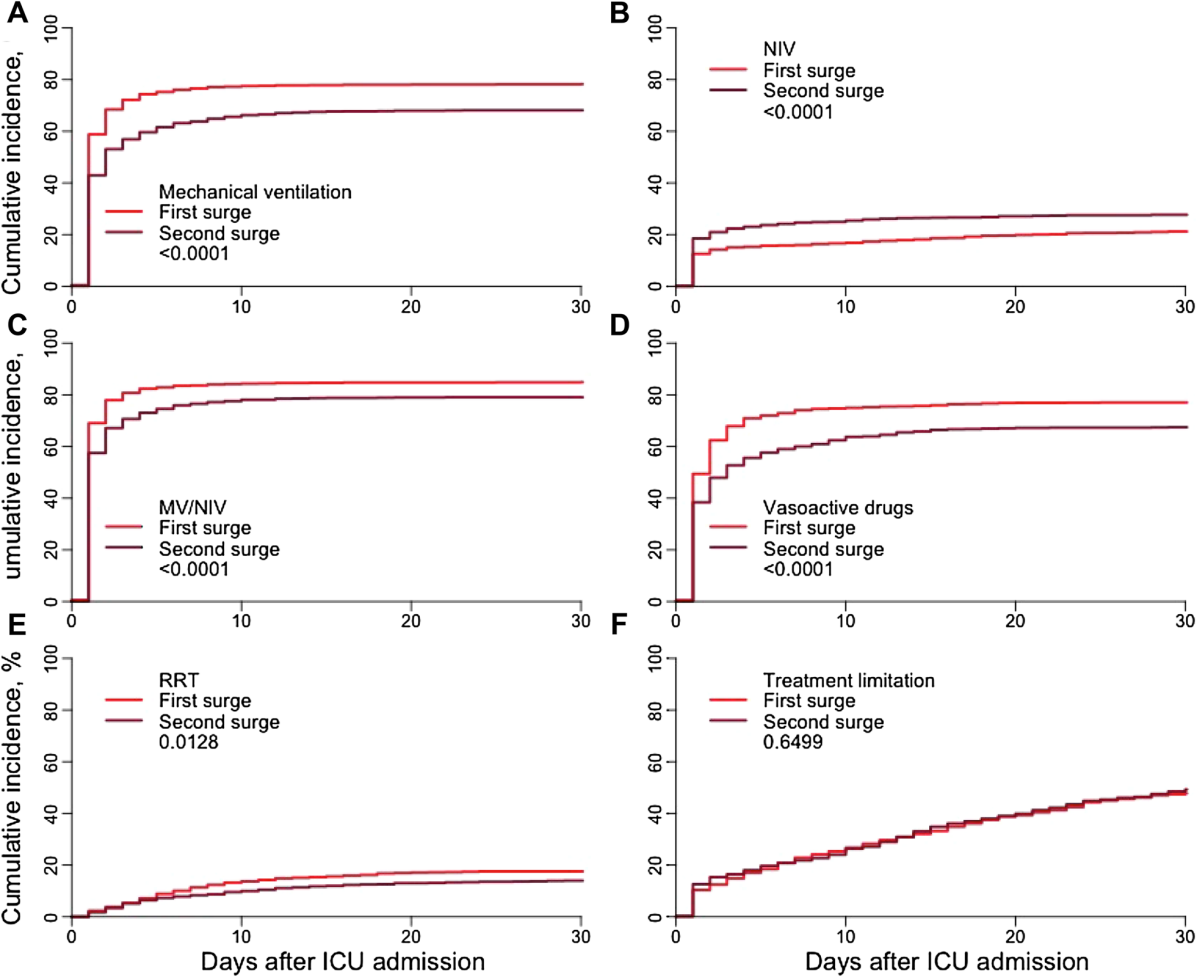
Cumulative incidences for **a** mechanical ventilation (MV), **b** non-invasive ventilation (NIV), **c** combined MV and NIV, **d** vasoactive drugs, **e** renal replacement therapy (RRT), and **f** treatment limitation during the first and the second surge

During the second surge of the pandemic, management had changed when compared with the first surge of the pandemic (Table 2). Patients were intubated for mechanical ventilation less frequently and if mechanical ventilation was performed, intubation took place later. By contrast, non-invasive ventilation was performed more often. Prone positioning was equivalent during both periods. Renal replacement therapy and treatment with vasoactive substances were performed less frequently in the second surge. This is also illustrated by the time-to-event analysis as outlined in Fig. 1. Treatment with antibiotics decreased, while the treatment with corticosteroids increased. Of note, there was no difference in withholding or withdrawal of treatment.

**Table 2:** Management of patients during the first and second surge of the pandemic

	First surge (until 26 May 2020)	Second surge 1 September–31 December 2020	*p *value
Patients (*n*)	1327	1298	
*Time periods*			
Days with symptoms prior to hospital admission	7 (4–10)	7 (4–10)	0.35
Days in the hospital prior to ICU admission	2 (1–4)	2 (1–5)	< 0.0001
Length of ICU stay for patients discharged alive (days)	15 (6–29)	10 (5–20)	< 0.0001
*Respiratory management*			
Mechanical ventilation started on day 1	58%	42%	< 0.0001
*Mechanical ventilation*			
Cumulative incidence at day 15 Cumulative incidence at day 30	78% (76–80) 78% (76–80)	68% (65–70) 68% (66–71)	< 0.0001
*Non-invasive ventilation*			
Cumulative incidence at day 15 Cumulative incidence at day 30	19% (17–21) 21% (19–23)	27% (24–29) 28% (25–30)	0.0012
*Non-invasive or mechanical ventilation*			
Cumulative incidence at day 15 Cumulative incidence at day 30	85% (83–86) 85% (83–87)	79% (76–81) 79% (77–81)	0.0002
*Prone positioning*			
Cumulative incidence at day 15 Cumulative incidence at day 30	57% (54–60) 58% (54–61)	55% (52–59) 56% (53–60)	0.6074
*Further management*			
Vasoactive drugs	77%	67%	< 0.0001
Renal replacement therapy	18%	14%	0.0069
Corticosteroids	38%	93%	< 0.0001
Antibiotics	92%	88%	0.0009
*Withholding or Withdrawal of treatment modalities*			
Cumulative incidence at day 15 Cumulative incidence at day 30	29% (27–32) 37% (35–40)	30% (27–32) 37% (35–40)	0.86

**Fig. 2:**
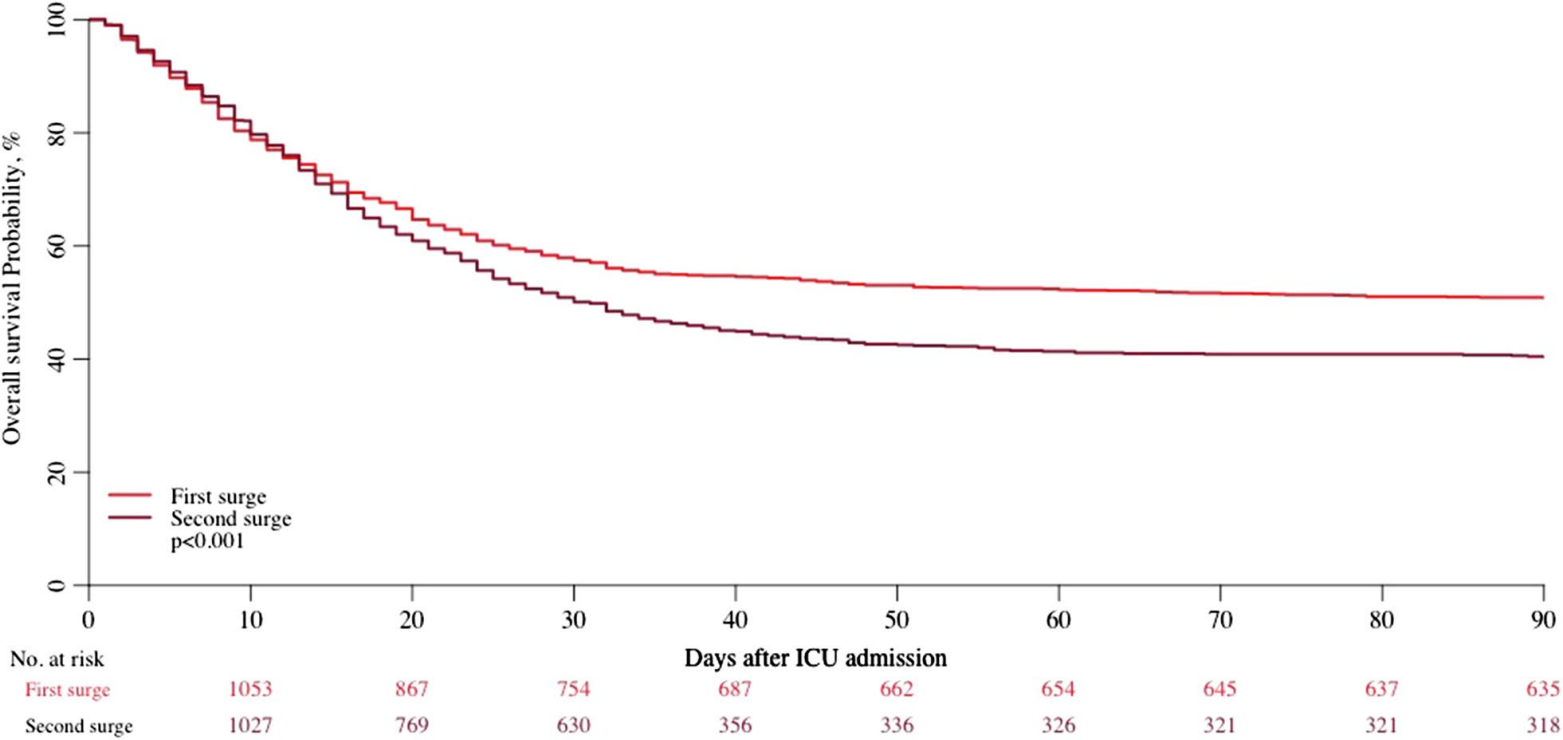
Kaplan–Meier curve until 90 days for patients admitted during the first and the second surge

**Fig. 3:**
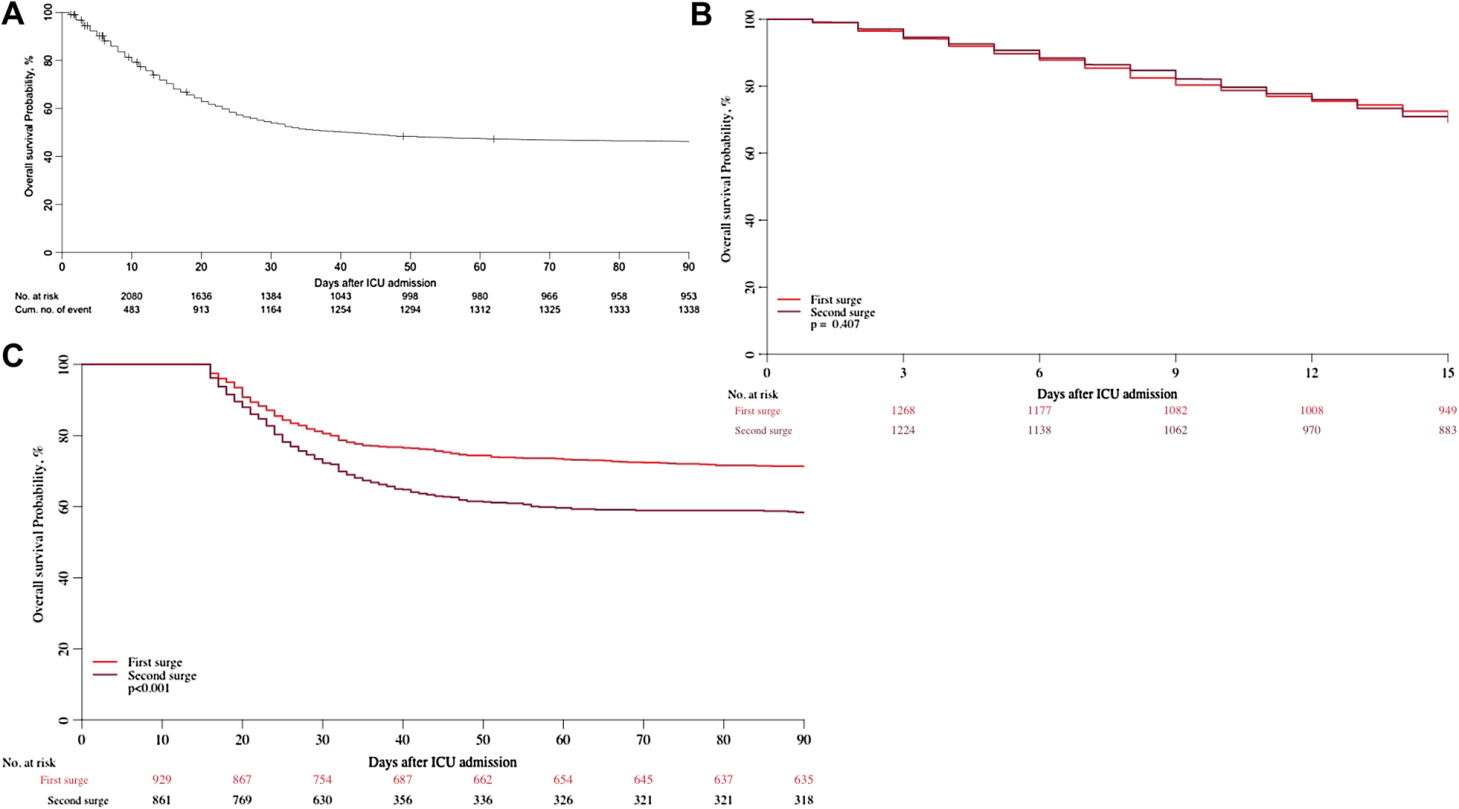
Overall survival probability plots **a** the entire cohort until day 90 following ICU admission; **b** a comparison between the two surges until day 15 after ICU admission; **c** landmark analysis comparing the two surges for patients alive at day 15 after ICU admission

We observed a lower rate of survival in the second surge of the pandemic (Table 3, Fig. 2, log rank: *p* < 0.001). Survival before 15 days was found to be similar for patients during the two surges; the unadjusted hazard ratio (HR) for survival before 15 days for the second versus first surge was 1.06 (95% CI 0.85–1.32, *p* = 0.62). Survival after day 15 was found to be worse for patients admitted to ICU during the second surge than for patients admitted during the first surge (Fig. 3). The unadjusted HR for survival after day 15 for the second surge versus first surge was 1.73 (95% CI 1.35–2.22, *p* < 0.001).

**Table 3:** Survival estimates after ICU admission during the two surges

	Surge 1	Surge 2
15-day survival	71.3% (95% CI 68.9–73.8)	69.3% (95% CI 66.8–71.9)
30-day survival	57.4% (95% CI 54.8–60.2)	50.1% (95% CI 47.4–52.9)
90-day survival	50.8% (95% CI 48.2–53.6)	40.3% (95% CI 37.5–43.4)

This survival difference persisted after adjustment for age, sex, PaO_2_/FiO_2_ ratio, other SOFA component, CFS, BMI, habitat and comorbidities (Table 4, HR > 1 represents increased mortality). Of note, important differences between the first and the second surge associated with worse outcome after day 15 are sex and kidney failure (Table 4B). All statistical details on this analysis with and without imputation for missing data for the overall survival probability analysis until day 90 are given in Additional file 7.

**Table 4:** Detailed analysis of factors associated with outcome in the treatment periods before and after 15 days after ICU admission. This landmark analysis allows differentiation of factors that are relevant for outcome earlier and later during the disease course

		HR (95% CI)	*p* value
*(A) Analysis of overall survival probability before day 15 (N* = *2625 patients)*			
Surge	Second versus first	1.04 (95% CI 0.83–1.3)	0.7293
Frailty (ref = Fit)	Vulnerable (CFS 4)	1.27 (95% CI 0.97–1.68)	0.0863
	Frail (CFS 5–8)	2.03 (95% CI 1.6–2.58)	< 0.0001
Age	One year increase	1.07 (95% CI 1.05–1.09)	< 0.0001
Habitat (ref = own home)	Other home with family or caregivers	1.36 (95% CI 1–1.84)	0.0506
	Nursing home	0.68 (95% CI 0.36–1.28)	0.2343
	Hospital ward	0.94 (95% CI 0.57–1.53)	0.7914
	Other/unknown	0.71 (95% CI 0.44–1.15)	0.1635
Sex	Female versus male	0.96 (95% CI 0.8–1.14)	0.6154
PaO_2_/FiO_2_ ratio	One point increase	1 (95% CI 1–1)	0.1628
Sofa_neuro	One point increase	1.15 (95% CI 1.06–1.25)	0.0009
Sofa_cardio	One point increase	1.01 (95% CI 0.95–1.07)	0.6976
Sofa_liver	One point increase	1.09 (95% CI 0.92–1.31)	0.3166
Sofa_coag	One point increase	1.17 (95% CI 1–1.37)	0.0499
Sofa_kidney	One point increase	1.31 (95% CI 1.19–1.43)	< 0.0001
BMI	One point increase	1.01 (95% CI 0.99–1.03)	0.2848
Diabetes (any type)	Yes versus no	1.13 (95% CI 0.93–1.39)	0.2196
Ischemic heart disease	Yes versus no	1.14 (95% CI 0.91–1.42)	0.2495
Renal insufficiency	Yes versus no	0.95 (95% CI 0.73–1.23)	0.6927
Arterial hypertension	Yes versus no	0.88 (95% CI 0.75–1.02)	0.0954
Pulmonary comorbidity	Yes versus no	1.07 (95% CI 0.9–1.28)	0.4122
Chronic heart failure	Yes versus no	1.1 (95% CI 0.84–1.43)	0.5031
*(B) Landmark analysis of overall survival probability after day 15 (patients alive 15 days after ICU admission and with follow-up* > 15 days—*N* = 1790)			
Surge	Second versus first	1.87 (95% CI 1.44–2.43)	< 0.0001
Frailty (ref = Fit)	Vulnerable (CFS 4)	1.24 (95% CI 0.91–1.69)	0.1661
	Frail (CFS 5–8)	1.53 (95% CI 1.11–2.11)	0.0088
Age	one year increase	1.04 (95% CI 1.02–1.06)	0.0008
Habitat (ref = own home)	Other home with family or caregivers	1.23 (95% CI 0.83–1.83)	0.3048
	Nursing home	0.43 (95% CI 0.17–1.09)	0.0738
	Hospital ward	0.98 (95% CI 0.7–1.37)	0.9088
	Other/unknown	0.86 (95% CI 0.55–1.32)	0.4847
Sex	Female versus male	0.75 (95% CI 0.6–0.93)	0.0088
PaO_2_/FiO_2_ ratio	One point increase	1 (95% CI 1–1)	0.3833
Sofa_neuro	One point increase	1.2 (95% CI 1.08–1.34)	0.0007
Sofa_cardio	One point increase	1.06 (95% CI 1–1.13)	0.0562
Sofa_liver	One point increase	0.94 (95% CI 0.77–1.14)	0.5151
Sofa_coag	One point increase	1.14 (95% CI 0.96–1.34)	0.1257
Sofa_kidney	One point increase	1.1 (95% CI 0.98–1.23)	0.1251
BMI	One point increase	0.99 (95% CI 0.97–1.02)	0.5460
Diabetes (any type)	Yes versus no	1.09 (95% CI 0.9–1.33)	0.3719
Ischemic heart disease	Yes versus no	0.77 (95% CI 0.57–1.05)	0.0956
Renal insufficiency	Yes versus no	1.39 (95% CI 1.02–1.9)	0.0366
Arterial hypertension	Yes versus no	0.92 (95% CI 0.77–1.09)	0.3095
Pulmonary comorbidity	Yes versus no	1.07 (95% CI 0.89–1.28)	0.4579
Chronic heart failure	Yes versus no	0.87 (95% CI 0.65–1.18)	0.3786

Also, the overall survival probability analysis confirmed increased mortality in the second surge. Furthermore, decreased survival has been confirmed by several sensitivity analyses for survival analysis (Additional file 8) and the adjusted survival models (model 2, HR > 1 indicates increased mortality in the second surge): patients’ age > 75: HR 1.35 (95% CI 1.12–1.62); patients with diabetes mellitus: HR 1.32 (95% CI 1.06–1.64); patients receiving mechanical ventilation: HR 1.45 (95% CI 1.21–1.73). Four countries were found to have recruited with asymmetric geometry across supplemental surges. However, a sensitivity analysis excluding these four countries confirmed previous findings (Additional file 9). In addition, Additional file 9 gives the sensitivity analysis excluding the first four weeks of each surge confirming previous findings.

## Discussion

In this study of patients aged 70 and above admitted with COVID-19 disease, we found a decrease in thirty-day survival from 57% in the first surge to 49% in the second surge, even after adjustment for important co-factors such as age, gender, SOFA score, comorbidities and frailty. The major differences between the two groups besides mortality were the reduction in the use of intubation and mechanical ventilation and its early use, reduced use of vasoactive drugs, increased use of non-invasive ventilation (NIV) and an increased use of corticosteroids during the second surge. Although management of patients changed, we cannot clearly attribute the change in outcome to a specific change in practice.

These findings are surprising, as the ICU community had gained experience in treating these patients during the first surge. Here, the initial very high reported [16] mortality was soon followed with a reduction in mortality towards the end of the first surge [17]. It was thought that in the second surge, the use of steroids in patients with severe respiratory distress, and the delay in intubation, following the use of NIV to its full potential would translate to better outcomes. However, our detailed analysis revealed that besides the treatment during the second surge, older age, male sex, increased frailty, increased SOFA score and chronic kidney disease were associated with poor outcome especially 15 days after ICU admission.

To date, there are only very few reports comparing the two surges in COVID-19 hospitalised patients. In a study from 955 US hospitals, researchers compared a trend analysis for the first surge: from 1 January to 30 April 2020 and 1 May to 30 June 2020. The overall hospital event rate for 30 day mortality or referral to hospice within 30 days fell from 16.56 to 9.29%, indicating improved outcome in the last period of the first surge suggesting a steep learning curve [18]. This has also been confirmed by a study from the UK in > 21,000 critical care patients with COVID-19 showing improved survival from March to June 2020 [19]. In another study looking at COVID-19 outcomes in hospitalised patients with rheumatic disease, outcomes from the first 90 days were compared to the following 90 days [20]. They found a reduced risk of hospitalisation and admission to an ICU in the late cohort, and also a fall in the risk of death (9.3% versus 4.5%).

Our patients were all treated in the ICU during both surges, and therefore, a comparison with these initial experiences from other patient groups is difficult. However, reports from Intensive Care National Audit and Research Centre (ICNARC) in the UK reveal valuable information in this respect. This national registry compared patients admitted before and after 1 September, 2020, and found a small increase in mortality in patients ventilated within the first 24 h from 46.6% in the first to 48.7% in the second cohort and a similar reduction in all patients discharged alive from the ICU [21]. Their results were also similar to our patient cohort in other respects, with considerably fewer patients receiving mechanical ventilation, (down from 72 to 50%) and more patients given basic respiratory support (from 25 to 47%). In addition, the duration of mechanical ventilation was shorter. Analogous to our results, they found that more patients had a low PaO_2_/FiO_2_ ratio at admission.

There are several possible reasons for the increased mortality seen in our study although we can only describe associations in this kind of study setting. While our data do not give a satisfactory explanation, it allows for several potential contributing factors to be discussed and to guide specific attention to differences occurring during the disease course, such as the increasing relevance of kidney failure and potential gender differences after day 15.

A worse outcome might have been caused by the increased length of time spent in other departments before ICU admission, resulting in patients deteriorating prior to eventual admission. This is supported by a decreased PaO_2_/FiO_2_ ratio seen at ICU admission in the second period, possibly suggesting more severe respiratory failure. This combined with a trend towards a reduction in the use of mechanical ventilation may not have been beneficial in this group of elderly critically ill patients, although this remains speculative. Although several studies and a meta-analysis suggested that timing of intubation may have no effect on mortality and morbidity in COVID-19 [22], this remains to be confirmed in elderly patients. Also failure of non-invasive ventilation with delayed intubation needs to better defined, with especially high mortality rates [23].

A similar rate of limitation of life-sustaining therapy was seen in the two cohorts, so this is unlikely to account for the difference in mortality. Two additional differences are a slight increase in age and frailty score in the second cohort, which could explain an increase in mortality; however, the difference in mortality remained even after adjustment for these factors.

Another possible explanation for the increased mortality, which cannot be ruled out, could be a reduction in quality of care, despite all dedicated efforts on the part of the staff, in the second compared to the first surge. When the second surge started, many hospitals and in particular ICUs had already been overstretched for half a year and were running well above their usual capacity. This had consequences for both the permanent staff who had been working increased hours over a long period of time, and also the continuous dilution of expertise as non-ICU personnel, both physicians and nurses were being brought in to work in ICUs. There has been great concern about the burden of work on the health of ICU workers [24], leading to fatigue and physical and mental health problems, which ultimately may affect quality of care. In the current survival analysis, survival differs from day 15 onwards and it is tempting to speculate that quality of care in particular had consequences for elderly patients with prolonged treatment duration.

Another important factor may be related to use of corticosteroids. In the second surge, 93% of our patients received corticosteroids, which is more than twice that found in the first cohort. It is well documented that steroids have potentially serious side effects in ICU patients. Steroids increase infection rate and hence mortality in patients admitted with influenza pneumonia [25] and thus could also increase the number of patients acquiring sepsis in the ICU. It was of interest to study the details in the supplementary appendix from the RECOVERY study where COVID-19 patients were randomised to receive corticosteroids [7]. Only a small number of patients requiring mechanical ventilation were over 70 years old. In a pre-specified analysis of the RECOVERY trial, there was no difference in mortality in patients above 70 years. Despite this, that landmark trial—among others—changed guidelines [26] and practice independent of age, in severely ill COVID-19 patients. Although the RECOVERY trial is a major achievement in these difficult times, some unanswered questions remained. For example, it was unclear whether patients with uncontrolled diabetes, acute delirium, underlying malignancy, immunosuppression, or other conditions in which corticosteroids might have harmful effects were included [8, 27].

Finally, development of COVID-19 mutations may change virulence and hence potentially lead to worse outcomes. It is well known that during the pandemic a new mutant virus emerged in Europe [28]. The clinical properties of this new strain are largely unknown as whole genome sequence studies have not been performed in large scale and ordinary COVID-19 testing does not differentiate mutant viruses from the original one. Such a cause for worse outcomes is uncertain but remains a theoretical possibility.

Another possible explanation could be selection bias creating differences between the cohort in the first and second surge. That patients are already selected before ICU admission is common, and many undergo formal and informal triage. Criteria for triage were extensively discussed from the beginning of the pandemic when some ICUs experienced a rapid overflow of patients. We do not have detailed insight in what happened before ICU admission in our study, as this was not a research question. There are, however, some casemix differences between the first and second surge, mainly connected to longer time in hospital before ICU admission during the second surge, and a decreased oxygen ratio at ICU admission. This indicates potential differences in initial treatment leading to changes in selection maybe connected with increased knowledge of the feasibility to treat some severe covid-19 patients outside the ICU [29].

Our study has further weaknesses mainly concerning the absence of details of variables that might account for our differences in outcome. There was no information about how steroids were administered, no control group of younger COVID-19 patients for comparison and there was no information about quality of care and the nurse-to-patient ratios as well as a measure of stress for personnel. Also, admission policies or local guideline changes were not recorded. The recording of treatment limitations is not without difficulties but important in ICU studies in elderly patients [30]. The reception of this might differ across study sites reflecting a wide heterogenicity. In addition, although we provided definition of the comorbidities, their characterisation do not provide in-detail characterisation and differ across studies in the literature. Also, data on anticoagulation, sedation practices and on lung-protective ventilation were not collected which might account for outcome differences. Another limitation is that we did not ask centres to monitor consecutive inclusion with a screening log, serving as proofs of consecutive recruitment and allowing generalizability. We have no proofs that all eligible patients in all centres have been included into the study. Our observations can only describe associations without ascribing causality; however, we have observed that an untargeted but consistent change in practice has changed the outcomes between the cohorts in the two surges.

## Conclusion

This is the first study in critically ill elderly ICU patients with COVID-19 infection that compares mortality data between the first and second surges of the pandemic. We have found an unexpected but significant rise in mortality in elderly COVID-19 patients treated in the ICU during the second surge. The cause of this rise is unknown but possible explanations have been discussed. Our main concern is whether the widespread changes in practice and treatment of COVID-19 between the two surges have contributed to this increased mortality in elderly patients. Further studies are urgently warranted to provide more evidence for current practice in elderly patients.

## Supplementary Information

**Additional file 1**. List of collaborators: COVIP-study; Description: List of COVIP study collaborators with affiliations.

**Additional file 2**. COVIP Country map; Distribution of study sites and included patients per country. The first number is the number of ICUs per country, the second the total number of included patients per country.

**Additional file 3**. Number of included patients: n (% within the wave) for the first ((until 26 May 2020) and second wave (1 September–31 December 2020) per country.

**Additional file 4**. Recruitment within the individual countries in relation to the start of the study.

**Additional file 5**. Consort flow chart illustrating screening and inclusion into the COVIP study

**Additional file 6**. A detailed definition of comorbidities and organ support.

**Additional file 7**. All statistical details on this analysis with and without imputation for missing data for the overall survival probability analysis until day 90 are

**Additional file 8**. Kaplan–Meier for different subgroups divided into the first and second wave with several sensitivity analyses for survival analysis and the adjusted survival models.

**Additional file 9**. Subgroup analysis for different subgroups with a sensitivity analysis excluding the first four weeks of each surge

## Declarations

### Availability of data and materials

Individual participant data that underlie the results reported in this article are available to investigators whose proposed use of the data has been approved by the COVIP steering committee.
