# Supplementary material for: Differences in mortality in critically ill elderly patients during the second COVID-19 surge in Europe
Source: Crit Care. 2021 Sep 23;25:344. doi: 10.1186/s13054-021-03739-7 (PMC8459701; doi:10.1186/s13054-021-03739-7)
Supplement: Supplementary file 1 — Additional file 1. List of collaborators: COVIP-study; Description: List of COVIP study collaborators with affiliations. [file 13054_2021_3739_MOESM1_ESM.docx]

**List of collaborators: COVIP-study**

| Hospital | City | ICU | Name |
| --- | --- | --- | --- |

**Austria**

| Medical University Graz | Graz | Allgemeine Medizin Intensivstation | Philipp Eller |
| --- | --- | --- | --- |
| Medical University Innsbruck | Innsbruck | Division of Intensive Care and Emergency Medicine, Department of Internal Medicine | Michael Joannidis |

**Belgium**

| Ziekenhuis Oost-Limburg | Genk | Department of Intensive Care | Dieter Mesotten |
| --- | --- | --- | --- |
| CHR Haute Senne | Soignies | Department of Intensive Care | Pascal Reper |
| Ghent University Hospital | Ghent | Department of Intensive Care | Sandra Oeyen |
| AZ Sint-Blasius | Dendermonde | Department of Intensive Care | Walter Swinnen |
| Clinique Saint Pierre Ottignies | Ottignies | Department of Intensive Care | Nicolas Serck |
| Universitair Ziekenhuis Brussel | Brussel | ICU UZB | Elisabeth Dewaele |

**Denmark**

| Herlev og Gentofte Hospital | Herlev | Intensiv Behandling | Helene Brix |
| --- | --- | --- | --- |
| Slagelse | Slagelse | Intensiv | Jens Brushoej |
| Regionshospitalet Horsens | Horsens | Intensiv | Pritpal Kumar |
| Odense University Hospital | Odense | Intensive Care Unit | Helene Korvenius Nedergaard |
| Sygehus Lillebælt | Kolding | Intensiv | Helene Korvenius Nedergaard |
| Regionshospitalet Viborg | Viborg | Intensiv | Ida Riise Balleby |
| Sygehus Sønderjylland | Aabenraa | Department of Anaesthesia and Intensive Care | Camilla Bundesen |
| Regionshospitalet Herning | Herning | Intensiv Afdeling | Maria Aagaard Hansen |
| Nordsjællands Hospital | Hillerød | Department of Anaesthesia and Intensive Care | Stine Uhrenholt |
| Regionshospitalet Randers | Randers | Intensiv | Helle Bundgaard |
| Aarhus University Hospital | Aarhus | Department of Intensive Care | Jesper Fjølner |

**England**

| Musgrove Park | Adult ICU | Taunton | Richard Innes |
| --- | --- | --- | --- |
| Princess Alexandra Hospital Harlow | Princess Alexandra Hospital ICU | Essex | James Gooch |
| Royal Papworth Hospital | Cardiothoracic Critical Care | Cambridge | Lenka Cagova |
| Royal Surrey Hospital NHS Foundation Trust | Intensive Care Unit | Guildford | Elizabeth Potter |
| Russells Hall | Russells Hall Anaes Dept | Dudley | Michael Reay |
| Tunbridge Wells Hospital | Tunbridge Wells Intensive Care/High Dependency Unit | Tunbridge Wells | Miriam Davey |
| Walsall Manor Hospital | Walsall ICU | Walsall | Mohammed Abdelshafy Abusayed |
| West Suffolk NHS Foundation Trust | Critical Care | Bury St Edmunds | Sally Humphreys |

**France**

| Hôpital Privé Claude Galien | Quincy sous Sénart | Medico-surgical ICU | Arnaud Galbois |
| --- | --- | --- | --- |
| Saint Antoine | Paris | Medical ICU | Bertrand Guidet |
| Hôpital Ambroise Paré | Boulogne Billancourt | Medico-urgical ICU | Cyril Charron |
| Hopital Européen Georges Pompidou | Paris | Medical ICU | Caroline Hauw Berlemont |
| CHU de Besançon | Besançon | Medico-surgical ICU | Guillaume Besch |
| Dieppe General Hospital | Dieppe | Medical ICU | Jean-Philippe Rigaud |
| CHU Amiens | Amiens | Medical ICU | Julien Maizel |
| Tenon | Paris | Medico-surgical ICU | Michel Djibré |
| Clinique Du Millenaire | Montpellier | Surgical ICU | Philippe Burtin |
| Marne La Vallee | Jossigny | Medico-surgical ICU | Pierre Garcon |
| CHU Lille | Lille | Medical ICU | Saad Nseir |
| CHU de Caen | Caen | Medical ICU | Xavier Valette |
| Compiegne Noyon Hospital | Compiegne | Medico-surgical ICU | Nica Alexandru |
| Cochin | Paris | Medical ICU | Nathalie Marin |
| CH Pau | Pau | Medico-surgical ICU | Marie Vaissiere |
| Victor Dupouy | Argenteuil | Medico-surgical ICU | Gaëtan Plantefeve |
| CH Saint Philibert | Lomme lez Lille | Medical ICU | Thierry Vanderlinden |
| Beaujon | Clichy | Medico-surgical ICU | Igor Jurcisin |
| Lariboisière | Paris | Medical ICU | Buno Megarbane |
| Lariboisière | Paris | Surgical ICU | Benjamin Glenn Chousterman |
| Saint-Louis | Paris | Surgical ICU | François Dépret |
| Saint Antoine | Paris | Surgical ICU | Marc Garnier |
| Louis Mourier | Colombes | Medico-surgical ICU | Sebastien Besset |
| Avicenne | Bobigny | Medico-surgical ICU | Johanna Oziel |
| Centre hospitalier de Versailles | Le Chesnay | Medico-surgical ICU | Alexis Ferre |
| Robert Debré | Paris | Pediatric ICU | Stéphane Dauger |
| Saint-Louis | Paris | Medical ICU | Guillaume Dumas |
| Sainte-Anne | Paris | Medico-surgical ICU | Bruno Goncalves |
| CHU de Besancon | Besançon | Medical ICU | Lucie Vettoretti |
| CH Dr SCHAFFNER, Reanimation polyvalente | Lens | Medico-surgical ICU | Didier Thevenin |

**Germany**

| Charité - Universitätsmedizin Berlin | Berlin | 44i | Stefan Schaller |
| --- | --- | --- | --- |
| Charité - Universitätsmedizin Berlin | Berlin | W1 | Stefan Schaller |
| Florence-Nightingale Krankenhaus | Duesseldorf | 32 | Muhammed Kurt |
| Kliniken Nordoberpfalz AG Klinikum Weiden | Weiden | Interdisziplinäre Intensivmedizin | Andreas Faltlhauser |
| Charité - Universitätsmedizin Berlin | Berlin | 8i | Stefan Schaller |
| Evangelisches Krankenhaus Düsseldorf | Düsseldorf | Intensivstation | Christian Meyer |
| Malteser Krankenhaus St. Franziskus Hospital | Flensburg | Intensivstation 1 | Milena Milovanovic |
| Uniklinik Schleswig Holstein Campus Kiel | Kiel | Internistische Intensivstation | Matthias Lutz |
| Johanna Etienne Krankenhaus | Neuss | Station 2 | Gonxhe Shala |
| Kliniken Maria Hilf | Mönchengladbach | Internistische Intensivstation I und II | Hendrik Haake |
| Krankenhaus Bethanien GmbH Solingen | Solingen | Intensivpflege Bethanien | Winfried Randerath |
| Uniklinik Düsseldorf | Düsseldorf | MX01 | Anselm Kunstein |
| University Hospital Würzburg | Würzburg |  | Patrick Meybohm |
| Charité - Universitätsmedizin Berlin | Berlin | 43i | Stefan Schaller |
| St Vincenz | Limburg | ICU | Stephan Steiner |
| University Hospital Ulm | Ulm | IOI-Interdisziplinäre Operative Intensivmedizin | Eberhard Barth |
| Marienhospital Aachen | Aachen | ITS | Tudor Poerner |
| University Hospital Leipzig / Klinik und Poliklinik für Anästhesiologie und Intensivtherapie | Leipzig | IOI (Interdisciplinary Operational/Surgical ICU) | Philipp Simon |
| Charité - Universitätsmedizin Berlin | Berlin | 203i | Marco Lorenz |
| Städtische Kliniken Mönchengladbach | Mönchengladbach | Interdisziplinäre Intensivstation | Zouhir Dindane |
| Charité - Universitätsmedizin Berlin | Berlin | 144i | Karl Friedrich Kuhn |
| Klinikum Darmstadt GmbH | Darmstadt | Interdiszipinaere Operative Intensivstation Klinik fuer Anaesthesiologie und operative Intensivmedizin | Martin Welte |
| Elisabeth-Krankenhaus Essen | Essen | Kardiologisch-internistische Intensivstation | Ingo Voigt |
| Klinikum Konstanz | Konstanz | I01 | Hans-Joachim Kabitz |
| Medical Center - University of Freiburg | Freiburg | Anaesthesiologiesche Intensivtherapiestation | Jakob Wollborn |
| St. Franziskus-Hospital Münster | Münster | Klinik für Anästhesie und operative Intensivmedizin | Ulrich Goebel |
| University Hospital Cologne | Cologne | Surgical ICU of the Department of Anesthesiology | Sandra Emily Stoll |
| University Hospital Duesseldorf | Duesseldorf | CIA1 | Detlef Kindgen-Milles |
| Essen University Hospital | Essen | Ana Int | Simon Dubler |
| University Hospital Duesseldorf | Düsseldorf | MI1/2 | Christian Jung |
| Rechts der Isar Technical University | Munich | ICU 1 | Kristina Fuest |
| Universitätsmedizin der Johannes Gutenberg-Universität Mainz | Mainz | Anästhesie-Intensivstation | Michael Schuster |

**Greece**

| GENERAL HOSPITAL OF LARISSA | LARISSA | ICU | Antonios Papadogoulas |
| --- | --- | --- | --- |
| General University Hospital of Patras | Patras | ΜΕΘ | Francesk Mulita |
| Sotiria Hospital National and Kapodistrian University of Athens | Athens | ICU 1st Pulmonary and Critical Care Medicine Dpt | Nikoletta Rovina |
| Ught Ahepa | Thessaloniki | Metha | Zoi Aidoni |
| UNIVERSITY HOSPITAL (ATTIKON) | HAIDARI | 2nd DEPARTMENT OF CRITICAL CARE | Evangelia Chrisanthopoulou |
| UNIVERSITY HOSPITAL OF HERAKLION | HERAKLION | ICU UNIVERSITY HOSPITAL OF HERAKLION | Eumorfia Kondili |
| UNIVERSITY HOSPITAL OF IOANNINA | IOANNINA | INTENSIVE CARE UNIT | Ioannis Andrianopoulos |

**Netherland**

| Alrijne Zorggroep | Leiderdorp | ICU Department | Martijn Groenendijk |
| --- | --- | --- | --- |
| Canisius Wilhelmina Hospital | Nijmegen | C38 | Mirjam Evers |
| Canisius Wilhelmlina Ziekenhuis | Nijmegen | ICU Department | Mirjam Evers |
| Diakonessenhuis Utrecht | Utrecht | Diakonessenhuis | Lenneke van Lelyveld-Haas |
| Haga Ziekenhuis | The Hague | ICU Haga | Iwan Meynaar |
| Medisch Spectrum Twente | Enschede | Intensive Care Center | Alexander Daniel Cornet |
| Radboudumc | Nijmegen | Intensive Care department Radboudumc | Marieke Zegers |
| University Medical Center Groningen | Groningen | Department of Critical Care | Willem Dieperink |
| University Medical Center Utrecht | Utrecht | Intensive Care | Dylan de Lange |
| Zuyderland mc | Heerlen | Intensive Care | Tom Dormans |

**Norway**

| Haugesund Hospital | Haugesund | ICU | Michael Hahn |
| --- | --- | --- | --- |
| Haukeland University Hospital | Bergen | KSK-ICU | Britt Sjøbøe |
| Kristiansund Hospital Helse Møre og Romsdal HF | Kristiansund | ICU | Hans Frank Strietzel |
| Oslo University Hospital | Oslo | Surgical ICU | Theresa Olasveengen |
| Oslo University Hospital Rikshospitalet Medical | Oslo | Department of Critical Care and Emergencies | Luis Romundstad |
| Ålesund Hospital | Ålesund | Dept. Anesthesia and Intensive Care Surgical ICU | Finn H. Andersen |

**Poland**

|  |  |  |  |
| --- | --- | --- | --- |
| Clinical Hospital Heliodor Święcicki Medical University of Karol Marcinkowski in Poznan | Poznan | Anesthesiology Intensive Therapy and Pain Treatment | Anna Kluzik |
| Infant Jesus Clinical Hospital Medical University of Warsaw | Warsaw | I Department of Anaesthesiology and Intensive Care | Paweł Zatorski |
| Jagiellonia University Hospital Cracow | Cracow |  | Tomasz Drygalski |
| Military Hospital | Krakow | ICU | Wojciech Szczeklik |
| Military Institute of Medicine | Warsaw | COVID-19 ICU | Jakub Klimkiewicz |
| Pomeranian Medical University | szczecin | ICU | Joanna Solek-pastuszka |
| Provincial Specialist Hospital | Olsztyn | Department of Intensive Care | Dariusz Onichimowski |
| SPSK-1 | Lublin | II Klinika Anestezjologii i Intensywnej Terapii | Miroslaw Czuczwar |
| University Hospital in Opole | Opole | Department of Anesthesiology and Intensvie Care | Ryszard Gawda |
| Uniwersyteckie Centrum Kliniczne w Gdańsku | Gdańsk | Klinika Anestezjologii i Intensywnej Terapii | Jan Stefaniak |
| Voivodship Hospital in Poznan | Poznan | Intensive Care Unit | Karina Stefanska-Wronka |
| ZDROWIE Sp. z o.o. | Kwidzyn | Oddział Anestezjologii i Intensywnej Terapii | Ewa Zabul |

**Portugal**

| Centro Hospitalar de Tondela-Viseu EPE | Viseu | Unidade de Cuidados Intensivos Polivalente | Ana Isabel Pinho Oliveira |
| --- | --- | --- | --- |
| Centro Hospitalar do Médio Tejo | Abrantes | Serviço de Medicina Intensiva | Rui Assis |
| Centro Hospitalar e Universitário São João | Porto | Infectious Diseases ICU | Maria de Lurdes Campos Santos |
| Centro Hospitalar Tráz os Montes e Alto Dour | Vila Real | D | Henrique Santos |
| Curry Cabral Hospital | Lisbon | UCIP | Filipe Sousa Cardoso |
| Hospital de Beatriz Ângelo | Loures | Serviço de Medicina Intensiva | André Gordinho |

**Spain**

| Clínico Universitario Lozano-Blesa | Zaragoza | Unidad de Cuidados Intensivos | Maria José Arche Banzo |
| --- | --- | --- | --- |
| Clínico Universitario Lozano-Blesa | Zaragoza | Unidad de Cuidados Intensivos | Begoña Zalba-Etayo |
| COMPLEJO ASISTENCIAL DE SEGOVIA | SEGOVIA | UCI SEGOVIA | Patricia Jimeno Cubero |
| Complexo Hospitalario Universitario Ourense | Ourense | UCI CHUO | Jesús Priego |
| Corporació Sanitària Parc Taulí | Sabadell | Parc Taulí | Gemma Gomà |
| Germans Trias i Pujol | Badalona | General ICU | Teresa Maria Tomasa-Irriguible |
| H. Universitari i Politècnic La Fe | Valencia | General ICU | Susana Sancho |
| Hospital Alvaro Cunqueiro | Vigo | Servicio de Medicina Intensiva CHUVI | Aida Fernández Ferreira |
| Hospital de Tortosa Verge de la Cinta | Tortosa | Unidad de Cuidados Intensivos | Eric Mayor Vázquez |
| Hospital General Universitario de Albacete | Albacete | aapm111 | Ángela Prado Mira |
| Hospital Universitari Sagrat Cor | Barcelona | ICU | Mercedes Ibarz |
| Hospital Universitario de Burgos | Burgos | UCI Burgos | David Iglesias |
| Hospital Universitario de Getafe | Getafe | Getafe | Susana Arias-Rivera |
| Hospital Universitario de Getafe | Getafe | Medical-Surgical ICU | Fernando Frutos-Vivar |
| Hospital universitario Rey Juan Carlos | Mostoles | Cuidados intensivos | Sonia Lopez-Cuenca |
| Hospital Universitario Rio Hortega | Valladolid | Reanimación Quirurgica | Cesar Aldecoa |
| Hospital Universitario Río Hortega | Valladolid | Servicio de Medicina Intensiva - Unidad 1 | David Perez-Torres |
| Hospital Universitario Río Hortega | Valladolid | Servicio de Medicina Intensiva - Unidad 2 | Isabel Canas-Perez |
| Hospital Universitario Río Hortega | Valladolid | Servicio de Medicina Intensiva - Unidad 3 | Luis Tamayo-Lomas |
| Hospital Universitario Río Hortega | Valladolid | Servicio de Medicina Intensiva - Unidad 4 | Cristina Diaz-Rodriguez |
| Miguel Servet University Hospital | Zaragoza | Servicio de Medicina Intensiva | Pablo Ruiz de Gopegui |

**Switzerland**

| Centre Hospitalier Universitaire Vaudois | Lausanne | Service de Médecine Intensive Adulte (SMIA) | Nawfel Ben-Hamouda |
| --- | --- | --- | --- |
| Clinica Luganese Moncucco | Lugano | Servizio di anestesia e rianimazione | Andrea Roberti |
| Fribourg Hospital | Fribourg | Intensive Care Unit | Yvan Fleury |
| Geneva University Hospitals | Geneva | Department of Acute Medicine | Nour Abidi |
| Inselspital Bern | Bern | Universitätsklinik für Intensivmedizin | Joerg C. Schefold |
| Kantonspital Thurgau Frauenfeld | Frauenfeld | Institut für Anästhesiologie und Intensivmedizin | Ivan Chau |
| Kantonspital Thurgau Frauenfeld | Frauenfeld | Institut für Anästhesiologie und Intensivmedizin | Alexander Dullenkopf |

**Wales**

| Glan Clwyd Hospital | Bodelwyddan | Critical Care Unit | Richard Pugh |
| --- | --- | --- | --- |
| Wrexham Maelor Hospital | Wrexham | Critical Care | Sara Smuts |
