# Supplementary material for: Differences in mortality in critically ill elderly patients during the second COVID-19 surge in Europe
Source: Crit Care. 2021 Sep 23;25:344. doi: 10.1186/s13054-021-03739-7 (PMC8459701; doi:10.1186/s13054-021-03739-7)
Supplement: Supplementary file 2 — Additional file 2. COVIP Country map; Distribution of study sites and included patients per country. The first number is the number of ICUs per country, the second the total number of included patients per country. [file 13054_2021_3739_MOESM2_ESM.pptx]

## Slide 1
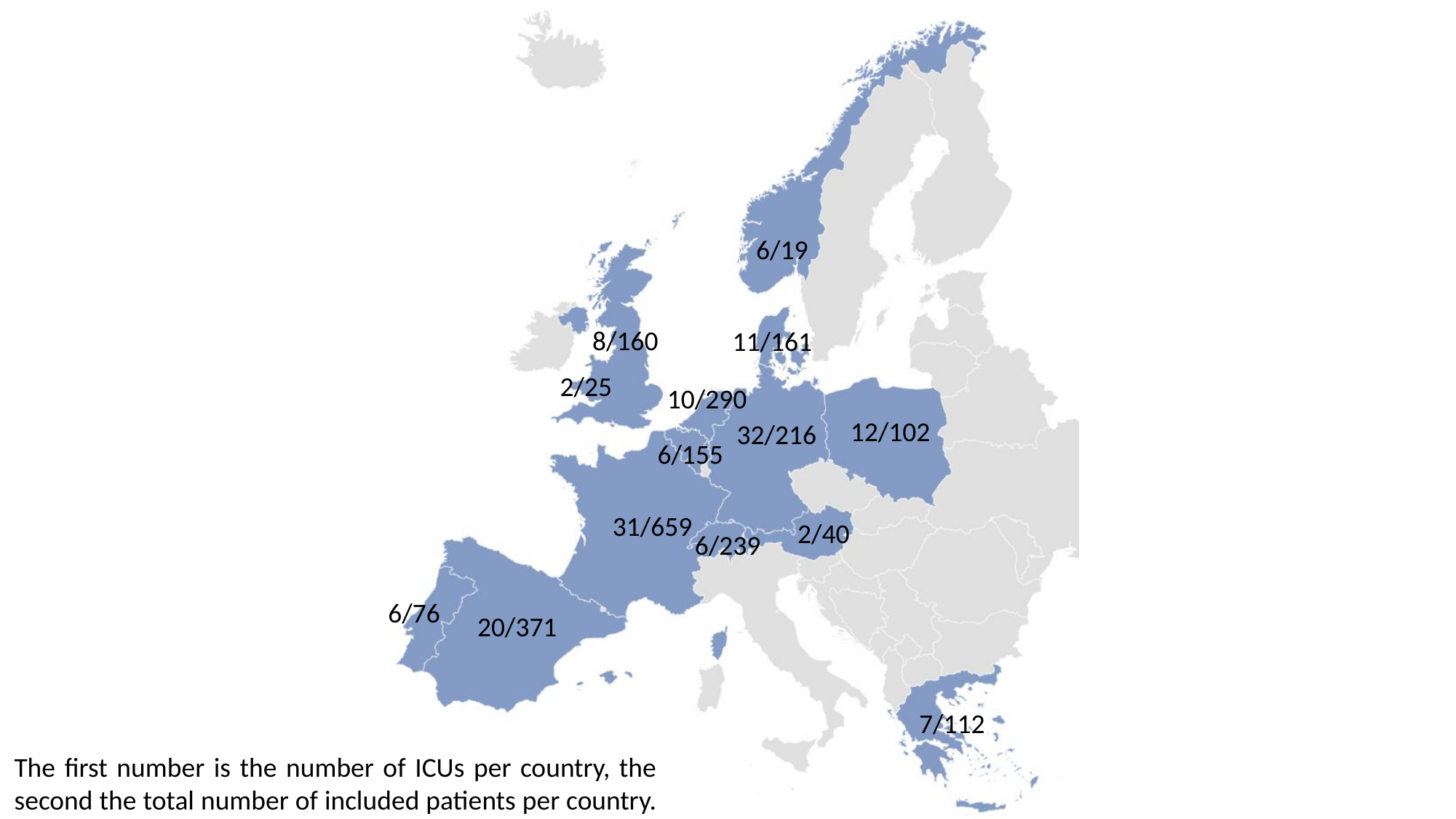

6/19
8/160
11/161
2/25
10/290
12/102
32/216
6/155
31/659
2/40
6/239
6/76
20/371
7/112
The first number is the number of ICUs per country, the second the total number of included patients per country.
