# Supplementary material for: Differences in mortality in critically ill elderly patients during the second COVID-19 surge in Europe
Source: Crit Care. 2021 Sep 23;25:344. doi: 10.1186/s13054-021-03739-7 (PMC8459701; doi:10.1186/s13054-021-03739-7)
Supplement: Supplementary file 3 — Additional file 3. Number of included patients: n (% within the wave) for the first ((until 26 May 2020) and second wave (1 September–31 December 2020) per country. [file 13054_2021_3739_MOESM3_ESM.docx]

|  | First wave  (until May 26th 2020)  Number of included patients: n (% within the wave) | Second wave  September 1^st^ – December 31^st^ 2020  Number of included patients: n (% within the wave) | Total number  N=2625 |
| --- | --- | --- | --- |
| Austria | 18 (1.4%) | 22 (1.7%) | 40 |
| Belgium | 78 (5.9%) | 77 (5.9%) | 155 |
| Denmark | 69 (5.2%) | 92 (7.1%) | 161 |
| England (GB) | 56 (4.2%) | 104 (8.0%) | 160 |
| France | 399 (30.1%) | 260 (20.0%) | 659 |
| Germany | 148 (11.2%) | 68 (5.2%) | 216 |
| Greece | 25 (1.9%) | 87 (6.7%) | 112 |
| Netherlands | 112 (8.4%) | 178 (13.7%) | 290 |
| Norway | 15 (1.1%) | 4 (0.3%) | 19 |
| Poland | 12 (0.9%) | 90 (6.9%) | 102 |
| Portugal | 41 (3.1%) | 35 (2.7%) | 76 |
| Spain | 206 (15.5%) | 165 (12.7%) | 371 |
| Switzerland | 130 (9.8%) | 109 (8.4%) | 239 |
| Wales (GB) | 18 (1.4%) | 7 (0.5%) | 25 |
