# Supplementary figures and images for: Differences in mortality in critically ill elderly patients during the second COVID-19 surge in Europe
Source: Crit Care. 2021 Sep 23;25:344. doi: 10.1186/s13054-021-03739-7 (PMC8459701; doi:10.1186/s13054-021-03739-7)

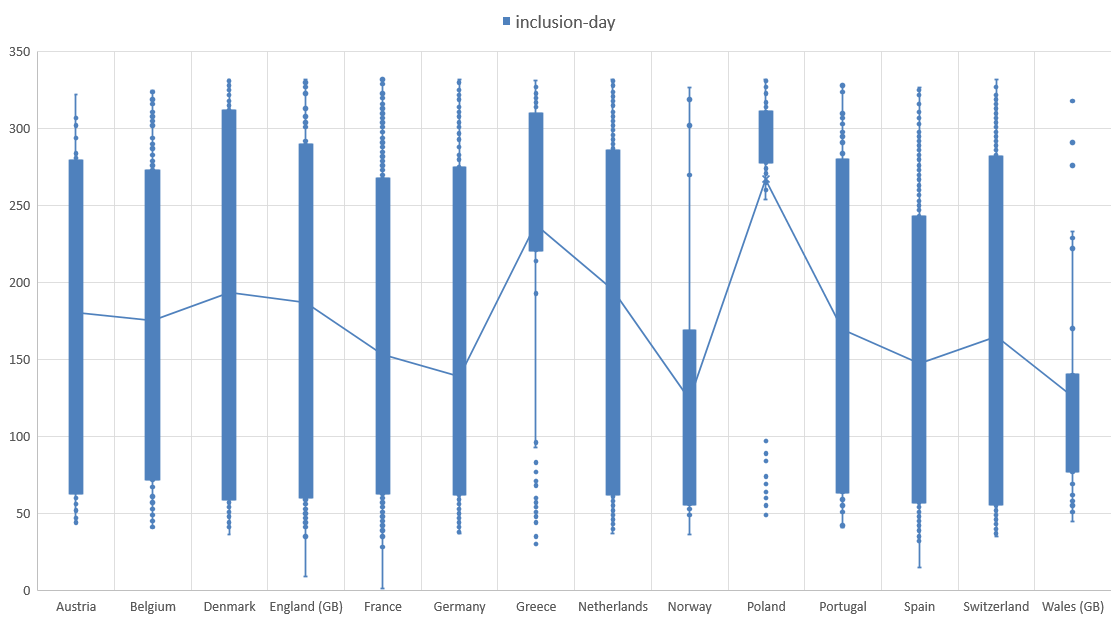

Supplement: Supplementary file 4 — Additional file 4. Recruitment within the individual countries in relation to the start of the study. [file 13054_2021_3739_MOESM4_ESM.png]

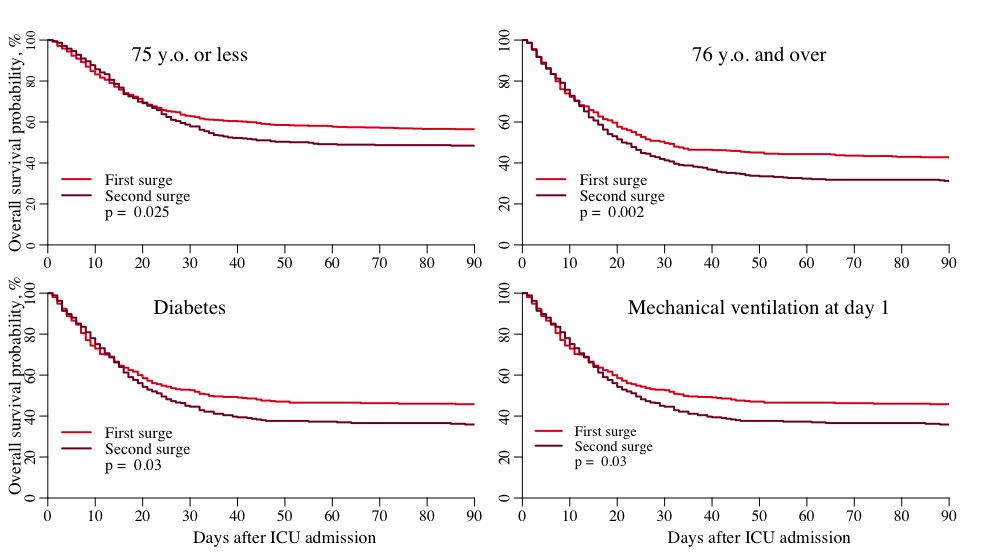

Supplement: Supplementary file 8 — Additional file 8. Kaplan–Meier for different subgroups divided into the first and second wave with several sensitivity analyses for survival analysis and the adjusted survival models. [file 13054_2021_3739_MOESM8_ESM.jpeg]
