# Supplementary material for: Differences in mortality in critically ill elderly patients during the second COVID-19 surge in Europe
Source: Crit Care. 2021 Sep 23;25:344. doi: 10.1186/s13054-021-03739-7 (PMC8459701; doi:10.1186/s13054-021-03739-7)
Supplement: Supplementary file 5 — Additional file 5. Consort flow chart illustrating screening and inclusion into the COVIP study [file 13054_2021_3739_MOESM5_ESM.pptx]

## Slide 1
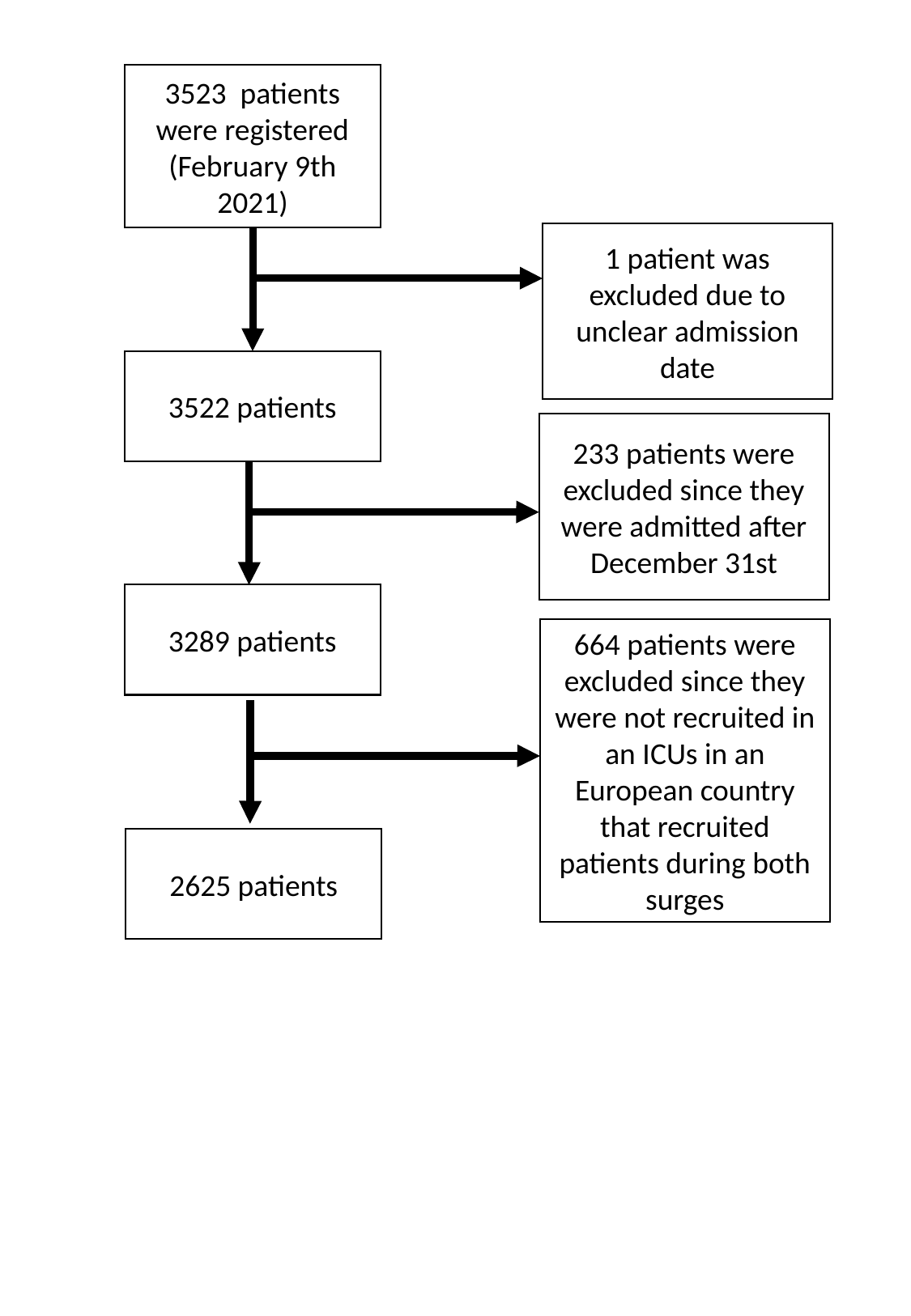

3523 patients were registered (February 9th 2021)
1 patient was excluded due to unclear admission date
3522 patients
233 patients were excluded since they were admitted after December 31st
3289 patients
664 patients were excluded since they were not recruited in an ICUs in an European country that recruited patients during both surges
2625 patients
