# Supplementary material for: Differences in mortality in critically ill elderly patients during the second COVID-19 surge in Europe
Source: Crit Care. 2021 Sep 23;25:344. doi: 10.1186/s13054-021-03739-7 (PMC8459701; doi:10.1186/s13054-021-03739-7)
Supplement: Supplementary file 6 — Additional file 6. A detailed definition of comorbidities and organ support. [file 13054_2021_3739_MOESM6_ESM.docx]

**Definitions**

**Definition of comorbidities:**

Diabetes mellitus: documented evidence of diabetes mellitus or reported by the patient or their relatives. Prescription of anti-diabetic medication or insulin on the drug chart.

Ischaemic heart disease: documented abnormal coronary angiography, known coronary artery disease, previous percutaneous coronary intervention (PCI) or coronary bypass surgery

Chronic renal failure: documented evidence of chronic renal insufficiency Grade 3 or higher, creatinine clearance <60ml/min or chronic dialysis

Arterial hypertension: documented evidence of any grade of chronic arterial hypertension or prescription of anti-hypertensive medication.

Pulmonary disease: documented evidence of or medication prescribed for chronic pulmonary disease of any aetiology (bronchial asthma, COPD, pulmonary fibrosis), or clinical or radiological signs of chronic pulmonary disease

Chronic heart failure: documented evidence of or medication prescribed for chronic heart failure of any aetiology or echocardiographic or radiological signs of chronic heart failure.

**Definition of organ support:**

Respiratory support: Mechanical ventilation (MV), non-invasive ventilation (NIV), prone position (passive), tracheostomy and extracorporeal membrane oxygenation (ECMO)

Circulatory support: use of inotropes or vasopressors

Renal support: use of continuous or intermittent renal replacement therapy
